# Amniotic Mesenchymal Stem Cells Enhance Wound Healing in Diabetic NOD/SCID Mice through High Angiogenic and Engraftment Capabilities

**DOI:** 10.1371/journal.pone.0041105

**Published:** 2012-07-17

**Authors:** Sung-Whan Kim, Hong-Zhe Zhang, Longzhe Guo, Jong-Min Kim, Moo Hyun Kim

**Affiliations:** 1 Regional Clinical Center, Dong-A University Hospital, Busan, South Korea; 2 Department of Cardiology, College of Medicine, Dong-A University, Busan, South Korea; 3 Department of Anatomy and Cell Biology, College of Medicine, Dong-A University, Busan, South Korea; Children’s Hospital Boston/Harvard Medical School, United States of America

## Abstract

Although human amniotic mesenchymal stem cells (AMMs) have been recognised as a promising stem cell resource, their therapeutic potential for wound healing has not been widely investigated. In this study, we evaluated the therapeutic potential of AMMs using a diabetic mouse wound model. Quantitative real-time PCR and ELISA results revealed that the angiogenic factors, IGF-1, EGF and IL-8 were markedly upregulated in AMMs when compared with adipose-derived mesenchymal stem cells (ADMs) and dermal fibroblasts. *In vitro* scratch wound assays also showed that AMM-derived conditioned media (CM) significantly accelerated wound closure. Diabetic mice were generated using streptozotocin and wounds were created by skin excision, followed by AMM transplantation. AMM transplantation significantly promoted wound healing and increased re-epithelialization and cellularity. Notably, transplanted AMMs exhibited high engraftment rates and expressed keratinocyte-specific proteins and cytokeratin in the wound area, indicating a direct contribution to cutaneous closure. Taken together, these data suggest that AMMs possess considerable therapeutic potential for chronic wounds through the secretion of angiogenic factors and enhanced engraftment/differentiation capabilities.

## Introduction

A chronic wound is defined as an open wound of the skin taking more than 8 weeks to heal. The impaired healing process is often associated with diabetic complications [1], [2], which can lead to severe outcomes including higher amputation rates and even death [3], [4]. Approximately 15% of diabetic patients suffer from non-healing chronic wounds [5]. Effective wound healing requires the highly organised integration of complex molecular and biological events including cell proliferation, migration and extra-cellular matrix (ECM) deposition. One of the major factors responsible for the appearance of chronic wounds is the impairment of cytokine release by local fibroblasts and inflammatory cells, which can result in reduced angiogenesis [5].

Mesenchymal stem cells (MSCs) derived from various tissues such as bone marrow, adipose tissues and cord blood have been reported to promote tissue repair. The possibility exists that human cord blood (CB) or AMMs could therefore be used as a convenient cell source for allogeneic cell transplantation [6], [7]. We previously demonstrated the efficacy of CB-derived MSCs for the improvement of peripheral circulation and rest pain in a clinical study [6]. Among the many types of MSCs, AMMs have particularly attractive merits for stem cell therapy. The use of AMMs usually incites less ethical concern than other fetal tissue-derived stem cells, partially due to the fact that they are abundantly available from waste placenta. In addition, they do not express major histo-compatibility complex (MHC) class II molecules and have lower expression of MHC class I than adult bone marrow (BM)-derived MSCs [8]. AMMs have also been reported to have high trans-differentiation and angio-vasculogenic potential in organ tissue [7], [9], [10].

Recently, the therapeutic effects of human BM, adipose and amniotic fluid-derived MSCs in wound healing models have been reported [11], [12], [13], [14]. Wu et al., demonstrated that BM-MSCs, by releasing high levels of angiogenic factors, significantly promoted wound healing [11]. However, the therapeutic properties of AMMs in chronic wounds remain to be fully elucidated. In the present study, we examined the angiogenic properties of AMMs and demonstrated their therapeutic effects for excisional skin wounds in diabetic mice compared with ADMs. We discovered several potential therapeutic advantages for AMMs, possibly attributable to the expression of multiple angiogenic factors.

## Methods

### Experimental Design

**Figure 1:**
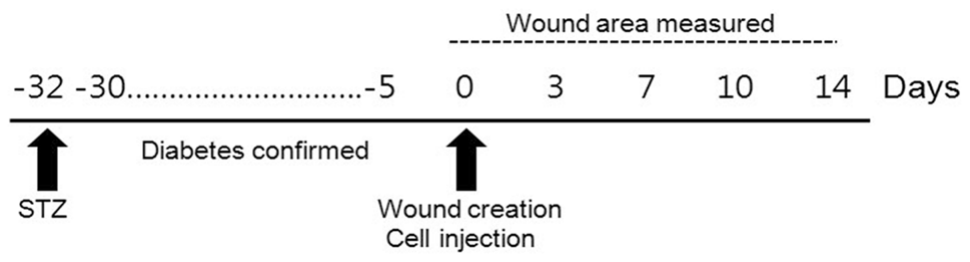
Experimental study design.

The overall experimental design is presented in Figure 1.

### Cell Culture

Normal human dermal fibroblasts (HDFs) and human umbilical vein endothelial cells (HUVECs) were purchased from the American Type Culture Collection (Manassas, VA, USA). Human adipose-derived MSCs (ADMs) and AMMs were purchased from Thermo Scientific Inc. (Rockford, IL, USA). HDFs, ADMs and AMMs were cultured in low-glucose DMEM (Gibco, Grand Island, NY, USA) supplemented with 10% FBS, 100 U/mL penicillin and 100 µg/mL streptomycin (Gibco). HUVECs were cultured in endothelial growth medium (EGM-2) (Lonza Walkersville, MD, USA). The average number of population doublings (PDs) was calculated as previously reported [15].

### Apoptosis Assay

Apoptotic cells was induced by serum deprivation (SD) for 6 h by modifying a previously reported method [16]. Apoptosis was measured using an Annexin V-FITC binding assay kit (Oncogene, San Diego, CA, USA) according to the manufacturer’s protocol. Apoptotic cells were analyzed using a FACScan (Becton Dickinson, San Jose, CA, USA) and CellQuest (Becton Dickinson) software.

### Flow Cytometry

ADMs and AMMs were passaged five times before re-suspension in PBS containing 1% BSA. Cells were incubated with FITC- or phycoerythrin (PE)-conjugated monoclonal antibodies specific for CD14, CD29, CD31, CD34, CD44, CD45, CD73, CD90, CD105 (endoglin), CD166, HLA-DR and rat anti-mouse IgG. All antibodies were purchased from Becton Dickinson or Biosciences Pharmingen (San Diego, CA, USA). Proper isotype-identical IgG served as a control. Cells were stained and then fixed in 2% paraformaldehyde before analysis on a flow cytometer (Becton Dickinson).

### Real-time PCR Analysis

A quantitative real-time (qRT-PCR) assay was conducted as described previously [17]. In brief, total RNA was isolated from cultured ADMs, AMMs, and HDFs passaged four times by using RNA-stat reagent (Iso-Tex Diagnostics, Friendswood, TX, USA), according to the manufacturer’s instructions. Extracted RNA was subsequently reverse-transcribed using Taqman Reverse Transcription Reagents (Applied Biosystems, Foster City, CA, USA), according to the manufacturer’s instruction. The synthesised cDNA was subjected to qRT-PCR using human-specific primers and probes. RNA levels were quantitatively assessed using an ABI PRISM 7000 Sequence Detection System (Applied Biosystems, Foster City, CA, USA). Relative mRNA expression normalised to GAPDH expression was calculated as described previously [18], [19]. We purchased all primer/probe sets from Applied Biosystems. Genes and catalogue numbers were as follows: *EGF*, Hs01099999-m1; *IGF-1*, Hs01547657-m1; *IL-8*, Hs00174103-m1; *GAPDH*, Hs99999905-m1.

### Conditioned Media (CM) Preparation

CMs were collected as described previously [20]. HDFs, ADMs, and AMMs (1 × 10^6^ cells each) were seeded into T-75 cell culture flasks and grown in normal medium or low-glucose DMEM (Gibco) containing 10% FBS, 100 U/mL penicillin and 100 µg/mL streptomycin (Gibco) for 48 h until the cells reached approximately 80% confluence. Culture media from each sample was then centrifuged at 1,000×*g* for 10 min and the supernatants were collected and used as CM for the study.

### Enzyme-linked Immunosorbent Assay (ELISA)

*In vitro* IL-8 and EGF secretion levels were assessed by using an IL-8 and EGF ELISA kit (Abcam, Cambridge, MA, USA) per supplier’s protocols. CMs were collected by the same method as previously described. Cell lysates (5×10^5^ cells) from each group were also collected in lysis buffer consisting of 10% glycerol, 25 mM Tris HCl, pH 7.5, 150 mM NaCl, 1% Triton X100, 5 mM EDTA and 1 mM EGTA.

### Scratch Wound Assay

The scratch wound assay was performed as described previously [20]. Briefly, HDFs and HUVECs were seeded to a final density of 1×10^5^ cells/well in 24-well culture plates coated with type I collagen (0.2 mg/mL) and incubated at 37°C in 5% CO_2_ for 24 h to create confluent mono-layers. The mono-layers were scratched using a sterile pipette tip and incubated with CM. Endothelial cell growth medium-2 (EGM-2; Lonza, Walkersville, MD) and low-glucose DMEM (Gibco) containing 10% FBS, 100 U/mL penicillin and 100 µg/mL streptomycin (Gibco) was used as control CM. To measure cell mobility, we obtained images at 5 random fields 6, 22, 48, and 72 h after scratching. The wound area was examined by the wound margin and calculated using the NIH Image program (http://rsb.info.nih.gov/nih-image/).

### Matrigel Tube Formation Assay

To examine the tube formation potential, HUVECs were seeded with each CM derived from the ADMs and AMMs, at a concentration of 1×10^4^ cells/well in basement membrane matrix gel (Matrigel, BD)-coated 2-well glass slides (NUNC). After 8 h of incubation, 10 fields from each sample were randomly photographed by inverted microscopy, and tube lengths were calculated by Image analysis system (Image J, Windows version; National Institutes of Health, USA).

### Generation of Type 1 Diabetes Animal Model

All experimental protocols were approved by the Dong-A University Institutional Animal Care and Use Committee and all procedures were performed in accordance with the Guide for the Care and Use of Laboratory Animals published by the US National Institutes of Health (NIH Publication No. 85-23, revised 1996). Male NOD/Severe Combined Immuno-Deficiency (SCID) mice (NOD.CB17-Prkdc^SCID^/J strain, The Jackson Laboratory, Bar Harbor, ME, USA) at 8–10 weeks old were intra-peritoneally injected with 45 mg/kg streptozotocin (STZ; Sigma, St. Louis, MO, USA) dissolved in 50 mM sterile citrate buffer (0.05 M sodium citrate, pH 4.5). STZ or citrate buffer (sham) was administered for 5 consecutive days during the first week of the study. Blood was collected from the tail vein 4 weeks after the injection and the blood glucose concentration was measured using a glucose analyser (Beckman Instrument, Palo Alto, CA, USA). Mice with blood glucose levels >280 mg/dL were considered diabetic and used for wound experiments.

### Full-thickness Excisional Wound Model and Cell Transplantation

Male NOD/SCID STZ-induced diabetic mice that were 12–14 weeks old and weighed 20–25 g were randomly divided into 4 groups: sham (control, *n* = 6), HDF-injected (HDF, *n* = 6), amniotic MSC-injected (AMM, *n* = 6), and adipose MSC-injected groups (ADM, *n* = 6). The excisional splinting model was generated as described previously [21]. There were no significant differences between the blood glucose levels of the AMM (445.6±96.7), HDF (459±110.1), and ADM groups (438.8±91.3) (*p* = 0.32, *p* = 0.28, each). In brief, all animals were anaesthetised by intra-peritoneal injection of 3.5% chloral hydrate (1 mL/100 g). Hair was removed from the dorsal surface, before two 6-mm full-thickness excision skin wounds were created on each side of the midline using a surgical punch. HDFs, AMMs, and ADMs (1 × 10^6^ cells suspended in 100 µL PBS) labelled with chloromethylbenzamido-1,1′-dioctadecyl-3,3,3′3′-tetramethylindo-carbocyanine (CM-Dil; Molecular Probes, Eugene, OR, USA), as described previously [22], were injected intra-dermally around the wound. In the control group, 100 µL PBS was injected intra-dermally around the wounds at the injection sites. A donut-shaped silicone splint (Grace Bio-Labs, Bend, OR, USA) was used, with the wounds centred within the splint. An immediate bonding adhesive (Krazy Glue; Elmer’s Inc., Columbus, OH, USA) was used to fix the splint to the skin followed by sutures to stabilise its position, and Tegaderm (3 M Health Care, St. Paul, MN, USA) was placed over the wounds.

### Wound Analysis

Photographs were obtained at days 0, 3, 7, 10 and 14. We defined wound closure as 100% re-epithelialization without drainage. The wound area was examined around the wound margin and calculated using NIH Image, blinded to grouping and treatment. The rate of wound closure was evaluated as follows: (original wound area – new wound area)/original wound area × 100. The splint was matched with the edge of the wound with the splint holes representing the original wound size.

### Histologic Analysis

The mice were euthanised at 14 and 28 days and skin wound samples were collected using a 10-mm biopsy punch. Tissue specimens were fixed with 4% paraformaldehyde for 1 day and embedded with OCT compound (Sakura Finetek USA, Torrance, CA, USA). Sections (10-µm thick) were stained with haematoxylin and eosin (H&E). Histologic scoring was performed in a blinded fashion, ranging from 1–9, according to the criteria presented in Table 1, modified from previous reports [21], [23]. For immuno-fluorescence studies, the tissue sections were incubated with sodium borohydride (1 mg/mL) to remove auto-fluorescence. Sections of each group were stained with primary mouse anti-cytokeratin (1∶200; Abcam, Cambridge, MA, USA) and secondary Cy2 (1∶400, Jackson ImmunoResearch, West Grove, PA, USA). Nuclei were stained with DAPI (Sigma). To identify human cells injected into mice, fluorescence in situ hybridization (FISH) was conducted with a Cy3-conjugated (Cambio, Cambridge, United Kingdom) human X chromosome probe as previously described [18]. Five fields from 5 tissue sections were randomly selected, with the number of positive cells in each field counted for quantification of differentiated and engrafted AMMs.

**Table 1:** Criteria for histological scores.

Score	Dermal and epidermal regeneration	Granulation tissue thickness	Angiogenesis (2weeks wounds only)
1∼3	Little epidermal and dermal organization	Thin granulation layer	Capillary density <300/mm^2^
4∼6	Moderate dermal and epidermal organization	Moderate granulation layer	Capillary density 300–500/mm^2^
7∼9	Complete remodeling of dermis and epidermis	Thick granulation layer	Capillary density >500/mm^2^

### Statistical Analysis

All data are presented as mean ± SEM. Statistical analyses were performed using Student’s *t*-test for comparisons between 2 groups and ANOVA with Bonferroni’s multiple comparison test by using SPSS v11.0. *p*<0.05 was considered statistically significant.

## Results

### Culture and Characteristics of AMMs

**Figure 2:**
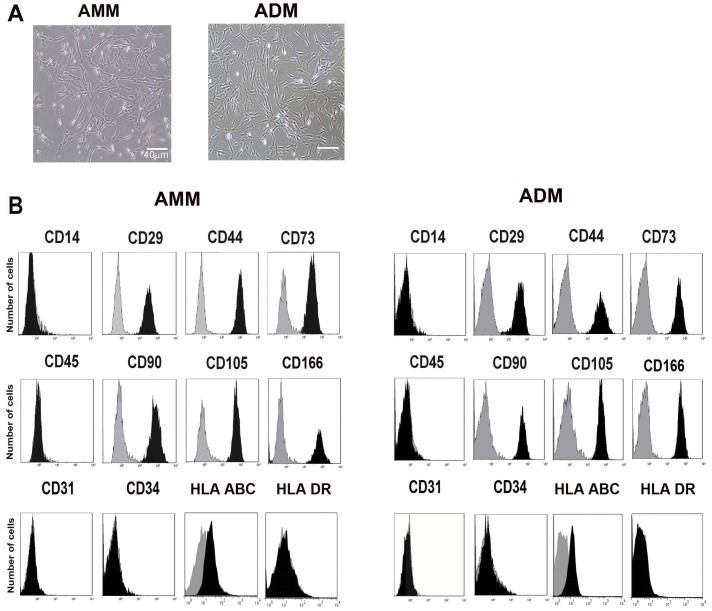
Characteristics of AMMs and ADMs. (A) Microscopic view of AMMs and ADMs (each passage 3). (B) Representative FACS surface markers of AMMs and ADMs. Isotype controls were overlaid in a gray color on each histogram for surface antigen tested.

Cultured AMMs exhibited spindle fibroblast-like morphology and showing high nucleus-to-cytoplasm ratios, similar to the morphology of ADMs (Figure 2A). FACS analysis of multiple surface epitopes showed that AMMs minimally expressed (<3%) haematopoietic cell markers (CD14, CD34 and CD45) and endothelial cell marker (CD31) (Figure 2B). In contrast, AMMs highly expressed CD29, CD44, CD73, CD90, CD105 and CD166, showing the specific characteristics of MSCs. These cells did not spontaneously differentiate and maintained their phenotypes during repeated subcultures during the cell expansion period.

### AMMs Express Enriched Angiogenic Genes and Proteins

**Figure 3:**
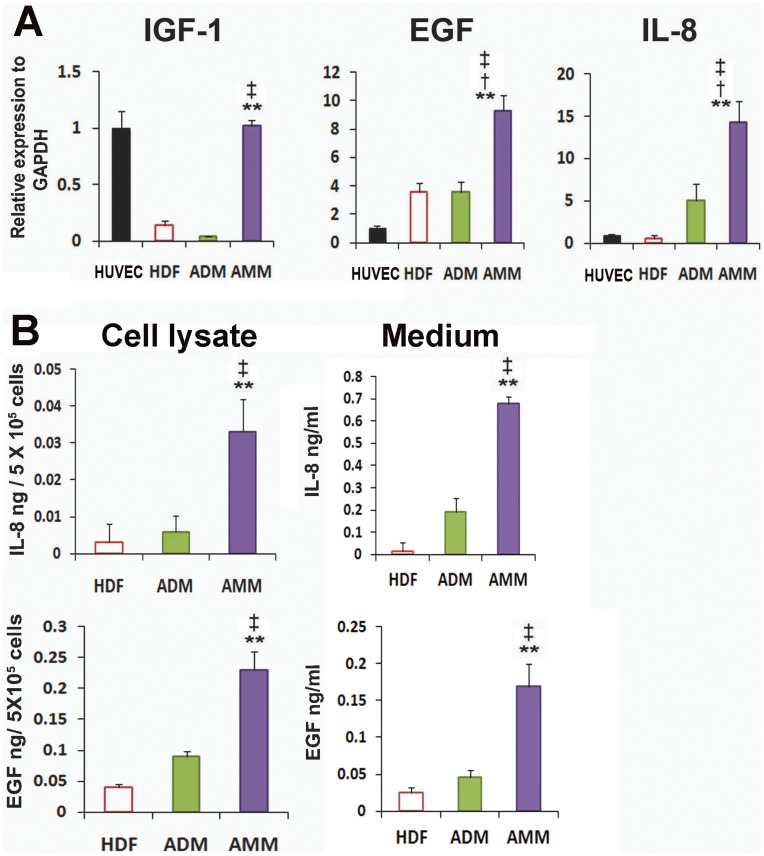
The expression patterns of angiogenic and and proteins. (A) qRT-PCR was performed to measure the gene expression levels of HUVECs, HDFs, ADMs, and AMMs. Various cytokines were up-regulated in the AMMs compared with other cell groups. Individual values were normalised to GAPDH. *n* = 4 per group. ** *p*<0.01 AMMs vs. ADMs, * *p*<0.05 AMMs vs. ADMs,^ ‡^ *p*<0.01 AMMs vs. HDFs, ^†^ *p*<0.01 AMMs vs. HUVs. Abbreviations: HUV, HUVECs. (B) ELISA for IL-8 and EGF in the cell lysate and supernatant from AMMs, ADMs and HDFs. The amount of IL-8 and IGF-1 were markedly higher in the AMMs than other ADMs and HDFs groups. *n* = 4 per group. ** *p*<0.01, * *p*<0.05, AMMs vs. ADMs,^ ‡^ *p*<0.01 AMMs vs. HDFs. (C) Comparison of the rate of cell apoptosis in response to SD. n = 5 per group. * *p*<0.05 AMMs vs. ADMs, ^†^ *p*<0.01 AMMs vs. HDFs.

To investigate the angiogenic properties of AMMs, we performed qRT-PCR analysis. We selected ADMs and HDFs as appropriate control groups, in order to specifically compare the properties of adult/fetal-derived stem cells with normal cells. AMMs showed significantly higher expression of the anti-apoptotic and angiogenic cytokine IGF-1(27-fold), when compared to ADMs (Figure 3A). In addition, EGF and IL-8 (known angiogenic factors that play pivotal roles in neovascularisation) were more highly expressed in AMMs compared to ADMs, HDFs and HUVECs (Figure 3A). To examine the quantity of IL-8 and EGF secreted by each cell type, we performed ELISA. ELISA detected significantly higher levels of IL-8 and EGF in AMM-lysate and supernatant compared to ADMs and HDF-lysate and supernatant (Figure 3B). To evaluate the cytoprotective effects of AMMs, apoptosis assay was performed. Apoptosis assay revealed that there were significantly fewer apoptotic cells in the AMMs compared with ADMs and HDFs (Figure 3C). Taken together, these data suggest that AMMs are a promising stem cell type enriched with angiogenic factors.

### Culture Media from AMMs Promotes Wound Closure and Tube Formation

**Figure 4:**
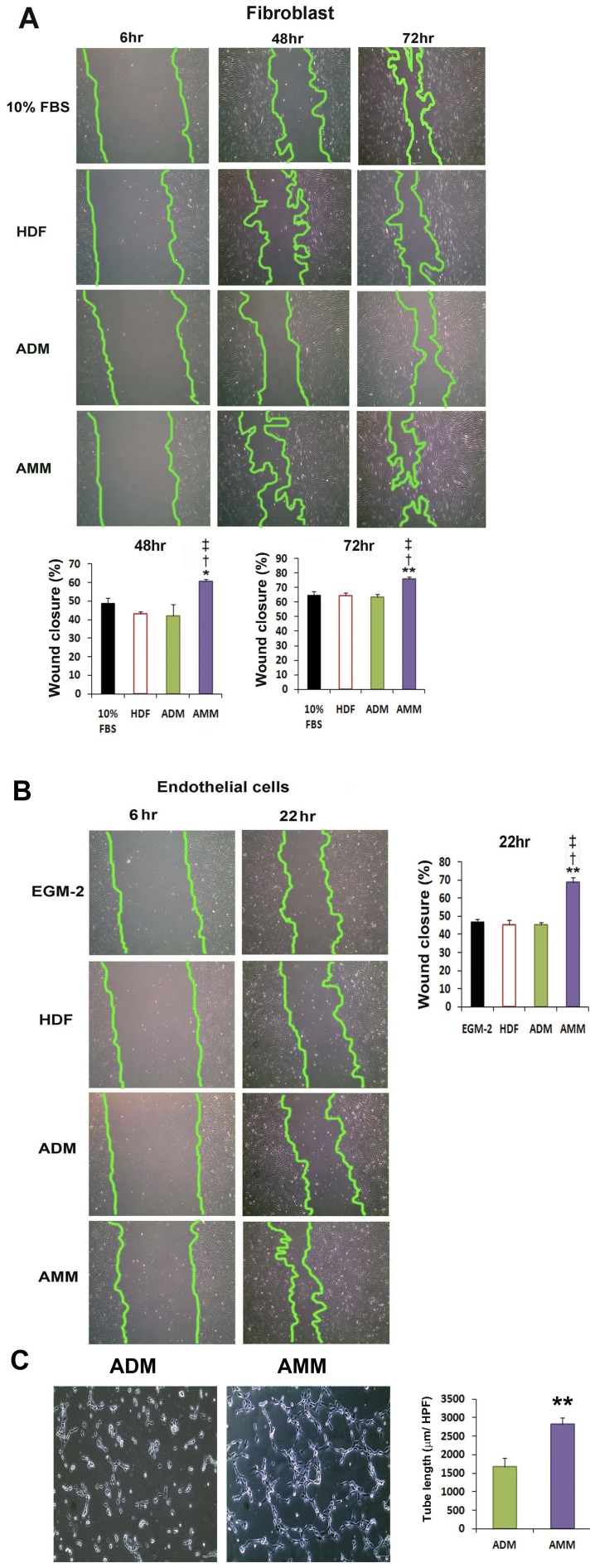
Scratch wound and Matrigel tube formation assays. (A) Representative photograph of fibroblast wound closure after incubation with CM. An in vitro wound healing assay showed that AMM CM strongly improved the fibroblast wound closure compared with the CM of HDF, ADMs, and the control group. (B) Representative photograph of wound closure of endothelial cells after incubation with CM. AMM CM significantly promotes the wound closure of endothelial cells compared with the CM of HUVECs, ADMs, and control. *n* = 4 per group. ** *p*<0.01 AMMs vs. ADMs, * *p*<0.05 AMMs vs. ADMs,^ ‡^ *p*<0.01 AMMs vs. HDFs, ^†^ *p*<0.01 AMMs vs. 10% FBS. (C) Representative photograph of Matrigel tube formation of endothelial cells after incubation with CM. *n* = 6 per group. ** *p*<0.01 AMMs vs. ADMs.

To examine whether proteins secreted from AMMs promote cell migration during wound closure, we performed a scratch wound assay. The scratch wound assay showed that AMM CM significantly increased the rate of fibroblast wound closure compared with that from ADM, HDF and control media at 48 h (60.6±1.2 vs 42±6.1; *p*<0.05, 43±1.4; *p*<0.01 and 49±2.6; *p*<0.05 respectively; *n* = 4) and 72 h (75.7±1.5 vs 63.4±1.8; *p*<0.01, 64.6±2.0; *p*<0.01 and 65±2.6; *p*<0.01 respectively; *n* = 4) (Figure 4A). In addition, AMMs CM markedly increased the rate of wound closure of endothelial cells compared with the ADMs, HDFs and control CM at 22 h (68.8±2.5 vs 45.1±1.4, 45.3±1.5, and 47.2±1.4, respectively; *n* = 4) (Figure 4B). In addition, AMM-CM induced significantly higher tube lengths (1.68-fold) as compared to ADM-CM (Figure 4C).

### AMMs Enhance Wound Healing

**Figure 5:**
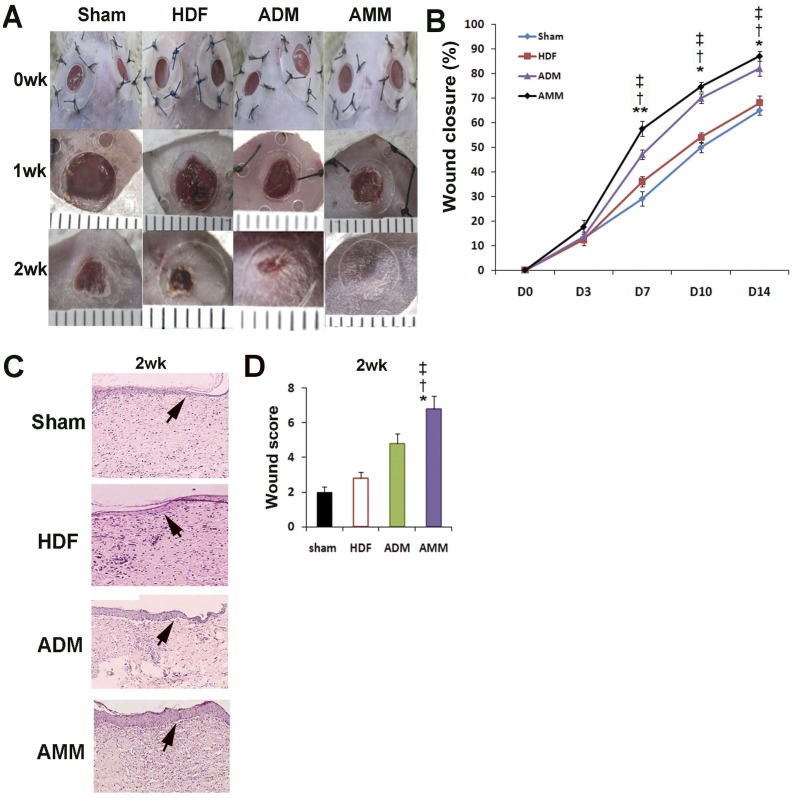
Effects of AMMs on the in vivo wound closure in mice model and histological analysis of wounds. (A) Representative photographs of the mouse excisional wound splinting model after transplantations of control vehicle medium (sham), HDFs, ADMs and AMMs at day 0, and 1 and 2 weeks. (B) Wound measurement of each group in STZ-diabetic mice. *n* = 6 per group. Abbreviations: wk, week(s). (C) Wound histological images by H&E staining. Cross-sectional wound areas were indicated by arrows. (D) Wound histological scores. *n* = 6 per group. ** *p*<0.01 AMMs vs. ADMs, * *p*<0.05 AMMs vs. ADMs,^ ‡^ *p*<0.01 AMMs vs. HDFs, ^†^ *p*<0.01 AMMs vs. sham.

To investigate the *in vivo* wound healing potential of AMMs, we induced diabetes using a NOD/SCID mouse model before creating excisional wounds. AMMs, ADMs and HDFs were then transplanted into dermis at a distance of 0.5 cm from the wounds. Wound healing results showed that AMM-treated wounds displayed accelerated wound healing at days 7, 10 and 14, compared with those treated with ADMs (day 7∶57.5±3.1% vs 40.1±2.2%; *p*<0.01, day 10∶74.5±2.0% vs 60.2±2.1%; *p*<0.05, day 14∶87.0±2.3% vs 74.3±3.3%; *p*<0.05; *n* = 6) and HDFs (Figure 5A and5B). Because the splints did not completely adhere to the mouse skins as a result of hair growth and movement, wound data after 14 days were not obtained.

Histologic wound scoring was conducted in a blinded fashion, ranging from 1–9 according to the criteria presented in Table 1, modified from previous reports [21], [23]. The wound area was examined around the wound margin and assessed using NIH Image. Histological analysis of wounds in the STZ-diabetic mice at Day 14 showed that AMM-treated wounds displayed enhanced re-epithelialisation when compared with ADM- and HDF-treated wounds (6.8±0.7 vs 4.8±0.6; *p*<0.05, 2.8±0.4; *p*<0.01; *n* = 6) (Figure 5C and 5D).

### AMMs Exhibit High Engraftment and Differentiation Rates

**Figure 6:**
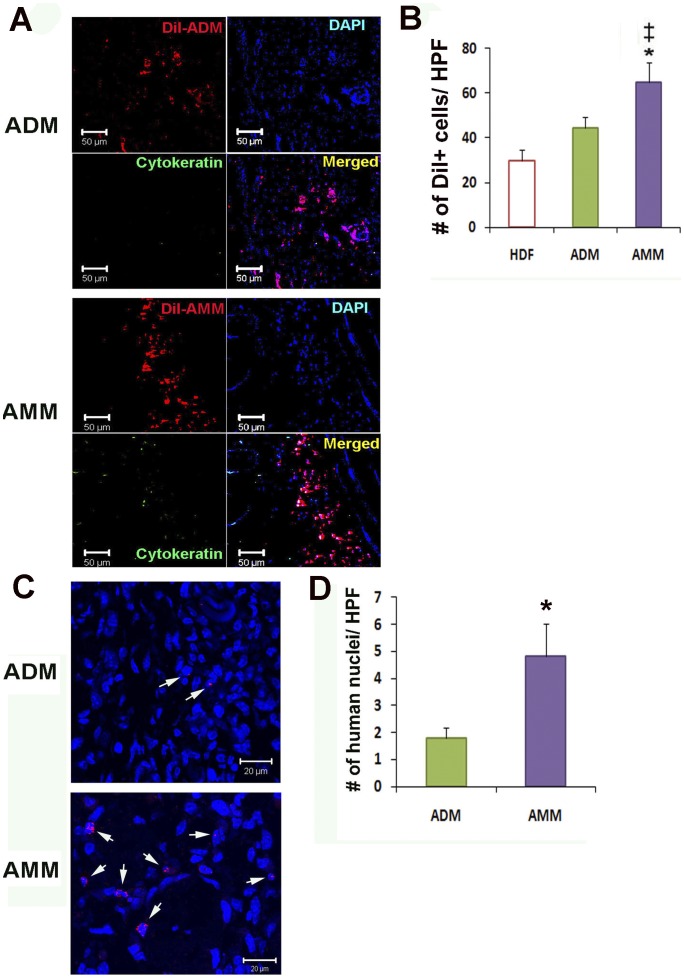
Engraftment potential of AMMs. (A) Representative photographs of localised Dil-labelled ADMs and AMMs. (B) Quantification of engrafted ADMs and AMMs. Dil-labelled cells (red) in the wound area were quantified by histological analysis 4 weeks after cell injection. Dil-labelled cells were injected into the peri-wound areas of NOD/SCID mice. Nuclei were stained with DAPI (blue). *n* = 5 per group. * *p*<0.05 AMMs vs. ADMs. (C) Fluorescent in situ hybridization on cell transplanted skin wound tissues. The cells (arrows) exhibited a fluorescent in situ hybridization signal (red) for human chromosome within the nuclei (arrows), suggesting engraftment of injected human cells. (D) Quantification of engrafted ADMs and AMMs by FISH analysis. Wound skin tissues were harvested 4 weeks after cell injection. *n* = 5 per group. * *p*<0.05 AMMs vs. ADMs.

To investigate the engraftment of transplanted cells in skin wounds, we performed immunohistochemistry in tissue specimens. The resulting analysis revealed that the engrafted cells in wounds was significantly higher in the AMM transplanted group than the ADM transplanted group (65.1±8.8 vs 44.6±4.5; *p*<0.05, *n* = 5) (Figure 6A). To confirm that engrafted cells were human-derived, we performed fluorescence in situ hybridization (FISH). These results also revealed increased numbers of human chromosome X probes in AMM-treated wounds when compared with ADM-treated wounds (Figure 6B).

**Figure 7:**
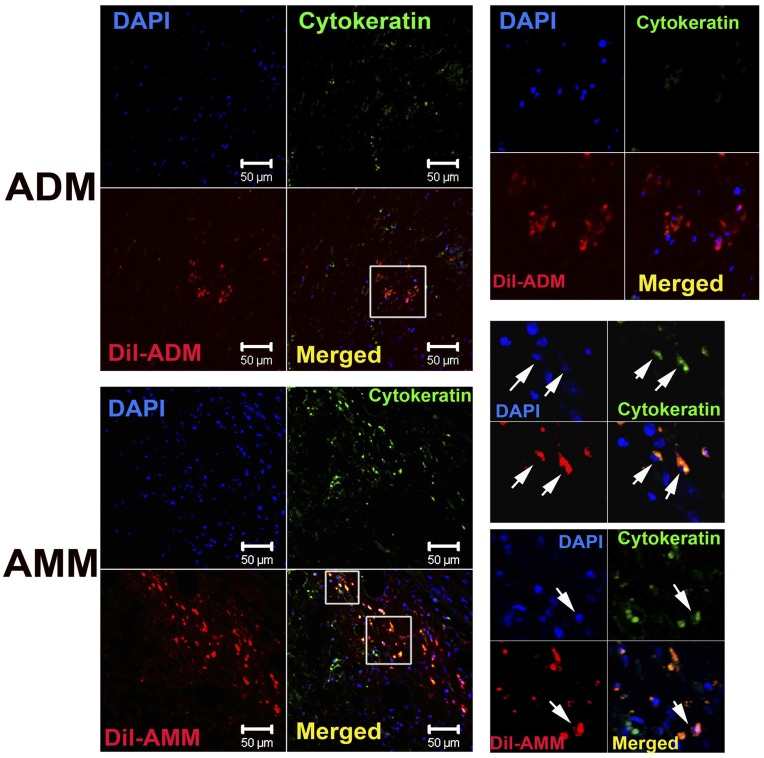
Differentiation potential of AMMs. (A) Representative photographs of cytokeratin-expressing ADMs and AMMs in vivo. Tissue sections from wound area 2 weeks after cell injection were stained with cytokeratin antibody (green). Confocal microscopy analysis showed that cytokeratin (green)-expressing AMMs (red) exist in the epidermis and dermis. Arrows indicate cytokeratin and Dil double-positive cells. (B) Quantification of cytokeratin (green)-positive transplanted (red) ADMs and AMMs. Arrows indicate cytokeratin positive Dil-labeled transplanted cells. ** *p*<0.01, *n* = 7.

To evaluate the keratinocyte differentiation capabilities of AMMs and ADMs, we performed immunostaining against the epithelial cell protein cytokeratin. We identified some keratinocyte like trans-differentiated cells around the epidermis of wound areras. At 28 days after Dil-labelled cell implantation, greater numbers of Dil and cytokeratin double-positive cells were observed in AMM-treated wounds than in ADM-treated wounds (7.6±1.5 vs 2.1±0.5; *p*<0.01, *n* = 7) (Figure 7A and 7B). These data suggest that AMMs possess high keratinocyte differentiation and cell survival potential in wounds.

## Discussion

In this study, we discovered that AMMs possess robust therapeutic properties for wound healing. The major findings of the present study are the following: (1) A higher expression and secretion of angiogenic genes and proteins when compared with ADMs; (2) AMM-derived CMs significantly enhanced cell migration; (3) Implantation of AMMs promoted wound healing in diabetic mice; (4) Transplanted AMMs highly engrafted and expressed keratinocyte specific proteins in wounds.

Vascular complications in diabetes and healing defects are associated with dysfunction and decreased numbers of endothelial progenitor cells (EPCs) [24], [25]. In addition, other cellular dysfunctions including inefficient reparative angiogenesis [26], abnormal control of cell survival [27] and impaired host signaling to the site of injury contribute to the delayed healing of diabetic wounds. Such impaired functions have been reported in cells derived from diabetic *db/db* mice [28]. We therefore postulated that the transplantation of amnion-, amniotic fluid- or cord blood-derived allogeneic stem or progenitor cells showing normal function and therapeutic potency for wound healing could be more effective than the use of patient-derived dysfunctional autologous stem/progenitor cells such as EPCs, ADMs or BM-MSCs.

However, concerns remain in regards to the immunogenicity of transplanted allogeneic cells. Studies have reported the absence of HLA-DR (MHC class II) and very low levels of HLA-ABC (MHC class I) expression in so-called ‘immune privileged’ foetal- or amnion-derived stem/progenitor cells resulting in low immunogenicity and immuno-modulatory effects after cell transplantation [8], [29]. Our flow cytometry results are in agreement with these findings and revealed that AMMs did not express class II MHC, a potentially favourable property for allogeneic cell therapy.

MSCs, which are so-called ‘stromal cells’, are well known to support the differentiation, growth and survival of haematopoietic stem cells (HSCs) in bone marrow through ECM signaling and various paracrine-secreted factors. Due to these activities, MSCs in the skin play a pivotal role in maintaining structural integrity and function, and have been found to. produce a wide variety of growth factors and cytokines [30]. Comparative analysis of BM-MSCs and HDFs shows that BM-MSCs secrete distinct levels of growth factors, thereby enhancing normal wound healing [31], [32].

Recently, we reported that AMMs have robust pro-angiogenic properties, showing better recovery of blood flow in an ischemic hindlimb model [10]. In that report, we identified AMM production of major angiogenic factors, including vascular endothelial growth factor (VEGF), hepatocyte growth factor (HGF) and angiopoietin. In this study, we identified further important angiogenic factors, namely EGF, IL-8 and IGF-1, that were also highly expressed in AMMs. EGF plays diverse roles in keratinocyte migration and angiogenesis [33], while IL-8 promotes skin re-epithelialisation by increasing keratinocyte proliferation [34] and migration [35]. Of the genes that were differentially upregulated, the very high expression of IGF-1 in AMMs when compared to ADMs, is particularly intriguing. IGF-1 was recently shown to have an important role in cell survival [36] and regeneration in various tissues [37], [38]. Thus, IGF-1 may be heavily involved in wound closure by significantly promoting growth of endothelial cells, dermal fibroblasts and keratinocytes. Taken together, these results suggest that elevated levels of angiogenic factors may be involved in determining favourable therapeutic effects in wound healing.

Because cell migration is a crucial event in cutaneous wound healing, the study of secreted factors that influence cell migration may help to generate an optimised therapeutic protocol for improved wound healing. Therefore, as an in vitro analysis of skin cell behaviour, scratch wound assays using HDFs and HUVECs were performed by modifying previously described methods [39]. In accordance with the gene expression data, AMM-derived culture media markedly affected the migration and proliferation of HDFs and HUVECs, when compared with ADMs, highlighting the therapeutic effects of crucial factors released by the transplanted MSCs. These results indicate differences in the cell migration and proliferation potential of various stem cell types. However, further study of primary human epidermal keratinocytes or co-culture of these different skin-derived cells may be important to validate in vitro models of skin cell behaviour.

Until recently, the trans-differentiation potential of bone marrow-derived MSCs into other lineages, such as cardiomyocytes (excluding bone, fat, and cartilage), remained controversial [40], [41]. This was until the trans-differentiation ability of AMMs into cardiomyocytes and keratinocytes was reported [7], [42], [43]. Additionally, attempts to implant allogeneic fibroblastshas resulted in low engraftment rates in wounds and caused increased inflammation [44], [45]. In agreement with these reports, HDF transplantation was found to have less therapeutic effects and was more likely to cause sudden death in mice compared with AMMs (data not shown). However, AMM injection resulted in no observable adverse effects and resulted in high wound closure rates when compared with HDF or ADM implantation. These data indicate that both the higher engraftment and trans-differentiation potential of AMMs induced by AMMs, including secretion of paracrine factors, may result in their therapeutic advantage.

In conclusion, this study demonstrated the robust beneficial effects of human AMMs on wound healing in a diabetic mouse model, via increased paracrine and engraftment effects. Therefore, AMMs may be a promising stem cell source for future cell based therapy.

### Study Limitations

We identified several limitations in our study. We were limited to the use of SCID mice, with possible negative implications for a normal wound healing setting. This may be because allogeneic cells might not survive and may have different effects in a different immunologic and inflammatory setting. We could also only perform a limited analysis regarding the effects on wound healing *in vivo*.
