# Correction: Amniotic Mesenchymal Stem Cells Enhance Wound Healing in Diabetic NOD/SCID Mice through High Angiogenic and Engraftment Capabilities

**DOI:** 10.1371/annotation/c39aa0ea-f26c-46ce-8213-94b533cd8d79

**Published:** 2012-10-15

**Authors:** Sung-Whan Kim, Hong-Zhe Zhang, Longzhe Guo, Jong-Min Kim, Moo Hyun Kim

A reference citation was erroneously omitted from the first sentence of the second paragraph of the Discussion. The reference is: Kang HJ, Kim JY, Lee HJ, Kim KH, Kim TY, Lee CS et al. Magnetic bionanoparticle enhances homing of endothelial progenitor cells in mouse hindlimb ischemia. Korean Circ J;42:390-396. 

