# Correction: Amniotic Mesenchymal Stem Cells Enhance Wound Healing in Diabetic NOD/SCID Mice through High Angiogenic and Engraftment Capabilities

**DOI:** 10.1371/annotation/23405c8e-1910-42c6-88eb-59e6fea5eafb

**Published:** 2012-10-15

**Authors:** Sung-Whan Kim, Hong-Zhe Zhang, Longzhe Guo, Jong-Min Kim, Moo Hyun Kim

A reference citation was erroneously omitted from the second sentence of the second paragraph of the Introduction. The reference is: Byun KH, Kim SW. Is stem cell-based therapy going on or out for cardiac disease? Korean Circ J 2009;39:87-92.

