# Correction: Amniotic Mesenchymal Stem Cells Enhance Wound Healing in Diabetic NOD/SCID Mice through High Angiogenic and Engraftment Capabilities

**DOI:** 10.1371/annotation/f6ebe3d3-ef7c-42ce-86fe-d5a661d7f67f

**Published:** 2012-10-15

**Authors:** Sung-Whan Kim, Hong-Zhe Zhang, Longzhe Guo, Jong-Min Kim, Moo Hyun Kim

There were errors in the published affiliations. The correct affiliations are:

Sung-Whan Kim^1,2^ Hong-Zhe Zhang^1,2^ Longzhe Guo^3^ Jong-Min Kim^4^ Moo Hyun Kim^1,2^

1 Regional Clinical Trial Center, Dong-A University Hospital, Busan, South Korea

2 Department of Cardiology, College of Medicine , Dong-A University, Busan , South Korea

3 Department of Cardiology, The Forth Hospital of Harbin Medical University, Harbin, China

4 Department of Anatomy and & Cell Biology and Mitochondria Hub Regulation Center, College of Medicine, Dong-A University, Busan, South Korea

